# Treatment of aneurysmal bone cysts using endoscopic curettage

**DOI:** 10.1186/s12891-018-2176-6

**Published:** 2018-07-27

**Authors:** Hisaki Aiba, Masaaki Kobayashi, Yuko Waguri-Nagaya, Hideyuki Goto, Jun Mizutani, Satoshi Yamada, Hideki Okamoto, Masahiro Nozaki, Hiroto Mitsui, Shinji Miwa, Makoto Kobayashi, Kojiro Endo, Shiro Saito, Taeko Goto, Takanobu Otsuka

**Affiliations:** 1 Department of Orthopedic Surgery, Nagoya City University Graduate School of Medical Sciences, 1 Kawasumi, Mizuho-cho, Mizuho-ku, Nagoya, 467-8601 Japan; 2 Department of Joint Surgery for Rheumatic Diseases, Nagoya City University Graduate School of Medical Sciences, 1 Kawasumi, Mizuho-cho, Mizuho-ku, Nagoya, 467-8601 Japan; 3 Department of Rehabilitation Medicine, Nagoya City University Graduate School of Medical Sciences, 1 Kawasumi, Mizuho-cho, Mizuho-ku, Nagoya, 467-8601 Japan; 4 Department of Radiology, Nagoya City University Graduate School of Medical Sciences, 1 Kawasumi, Mizuho-cho, Mizuho-ku, Nagoya, 467-8601 Japan; 5 Department of Orthopedic Surgery, Ogaki Municipal Hospital, 4-86 Minaminokawa-cho, Ogaki, 503-8502 Japan

**Keywords:** Endoscopy, Endoscopic curettage, Bone tumour, Aneurysmal bone cyst

## Abstract

**Background:**

Although aneurysmal bone cysts (ABCs) are benign tumours, they have the potential to be locally aggressive. Various treatment approaches, such as en bloc resection, open curettage, radiotherapy, sclerotherapy, and embolization have been proposed, but the most appropriate treatment should be selected after considering the risk of tumour recurrence and treatment complications. Endoscopic curettage (ESC) may be a less invasive alternative to open curettage for ABC treatment. We aimed to describe the use of ESC for the treatment of ABCs and to report our clinical outcomes, including the incidence rate of recurrence, radiological appearance at final follow-up, time to solid union, complications, and postoperative function.

**Methods:**

Between 1998 and 2015, 30 patients (18 men and 12 women; mean age, 17.4 years) underwent ESC for the treatment of primary ABCs at our hospital (mean postoperative follow-up, 55 months). ESC was performed under arthroscopic guidance for direct visualization, and curettage extended until normal bone was observed in the medullary cavity. To investigate bone healing after ESC, we evaluated the consolidation of cysts at the final evaluation (based on the modified Neer classification) and time to solid union after surgery, which was defined as sufficient cortical bone thickness to prevent fracture and allow physical activities.

**Results:**

Recurrence was identified in 3 cases (10%). Curative outcomes were obtained after repeated ESC or open curettage. A log-rank analysis indicated that age < 10 years (*p* = 0.004) and contact of the tumour with the physis (*p* = 0.01) increased the risk of tumour recurrence. Residual tumours were identified in 9 cases (30%); these lesions remained inactive over the extended follow-up period. The average time to solid union after endoscopic curettage was 3.2 months. Transient radial nerve palsy was identified in 1 case. Good postoperative functional recovery occurred in all cases.

**Conclusions:**

ESC is a minimally invasive technique for the treatment of ABCs, and the tumour recurrence rate is comparable to that of other standard procedures. However, the application of this method should be carefully considered, especially for patients < 10 years and when the tumour comes in contact with the physis.

**Electronic supplementary material:**

The online version of this article (10.1186/s12891-018-2176-6) contains supplementary material, which is available to authorized users.

## Background

Aneurysmal bone cysts (ABC) are expandable cavities that form within bone and are filled with blood and lined with proliferative fibroblasts, giant cells, and trabecular bone [1]. An ABC generally arises either as a primary neoplasm derived from translocation [2] or as a secondary lesion adjacent to osteoblastoma, giant cell tumours, or other types of tumours [3]. The goal of treatment is to stop the progression of the lesion, relieve pain, and prevent pathological fracture of the bone [4]. The following various treatment approaches have been proposed to meet these therapeutic goals: en bloc resection; curettage with or without burring or adjuvant therapy [5, 6]; radiotherapy [7]; curopsy, which is a novel biopsy technique introduced by Reddy et al. [8]; sclerotherapy [9]; and embolization [10]. Selection of the most appropriate treatment for any case depends on the risk of tumour recurrence and comorbidity associated with the treatment. In this study, we proposed the use of endoscopic curettage (ESC) as minimally invasive surgery for the treatment of ABCs and reviewed the surgical and clinical outcomes of this treatment approach.

## Methods

### Patient selection for ESC and relevant characteristics of the study group

**Fig. 1:**
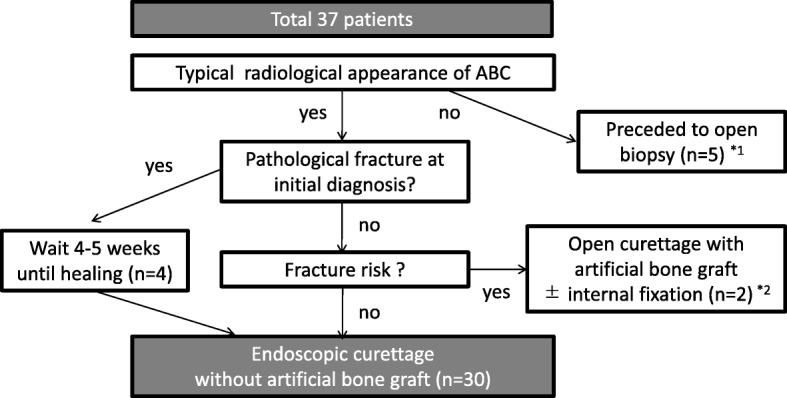
Flow diagram of patient selection for ESC treatment for ABC. For cases of atypical radiological appearance of an ABC, we proceeded with open biopsy to exclude the presence of a giant cell tumor, telangiectatic osteosarcoma, or other malignant bone tumor with a cyst. Moreover, to prevent femoral fracture after ESC, we proceeded with open curettage and artificial bone grafting, with or without internal fixation, for cases of large cystic lesions (lesion/cortex ratio > 2/3) located in the femoral trochanter (details provided in the Discussion). For ABCs at all other locations where a pathological fracture was visible at the time of the initial diagnosis, ESC was performed after bone union. Ultimately, 30 patients underwent ESC without an artificial bone graft. *1. In 5 cases, curettage with βTCP (OSferion; Olympus Co., Tokyo, Japan) was performed after intraoperative open biopsy for confirmation of ABC. This was because giant cell tumors were first suspected in 4 cases, and a cystic lesion that arose in an unusual location (clavicle) was not regarded as ABC according to the diagnosis indicated by preoperative imaging. *2: An ABC with a large cystic lesion (lesion/cortex ratio > 2/3) located in the femoral trochanter was regarded as a contraindication for ESC

From 1998 to 2014, 37 primary ABCs (37 patients) were diagnosed at our hospital, and histopathological confirmation was performed by our division of pathology. Among these, ESC was not performed for 7 cases after the initial clinical examination because of uncertainty regarding the diagnosis on imaging; therefore, open biopsy was indicated for exclusion of malignancy (*n* = 5) or the risk of pathological fracture after ESC (*n* = 2). The remaining 30 cases (18 men and 12 women; median age, 17.4 years; range, 5.8–40.0 years) were treated with ESC. For cases of pathological fracture before treatment, we waited 4–5 weeks until the cavities were closed because cortical continuity was necessary for irrigation of the cyst during surgery (Fig. 1). ABCs were located in tubular bones in 19 cases (12 humeri, 4 tibias, 2 femora, and 1 s metatarsal bone), in flat bones in 7 cases (3 ilia, 3 ischia, and 1 pubis), in short bones in 3 cases (2 calcanei and 1 os naviculare), and in a sesamoid bone in 1 case (patella). The mean maximum cyst diameter was 66.8 (standard deviation [SD], 32.9) mm. Patients were followed-up until bone healing; however, when the cyst did not consolidate over time, the patients were monitored for at least 2 years postoperatively. The mean overall follow-up period was 55.0 (SD, 34.3) months. Additional information regarding patient characteristics is available (Additional file 1).

### Surgical procedure

**Fig. 2:**
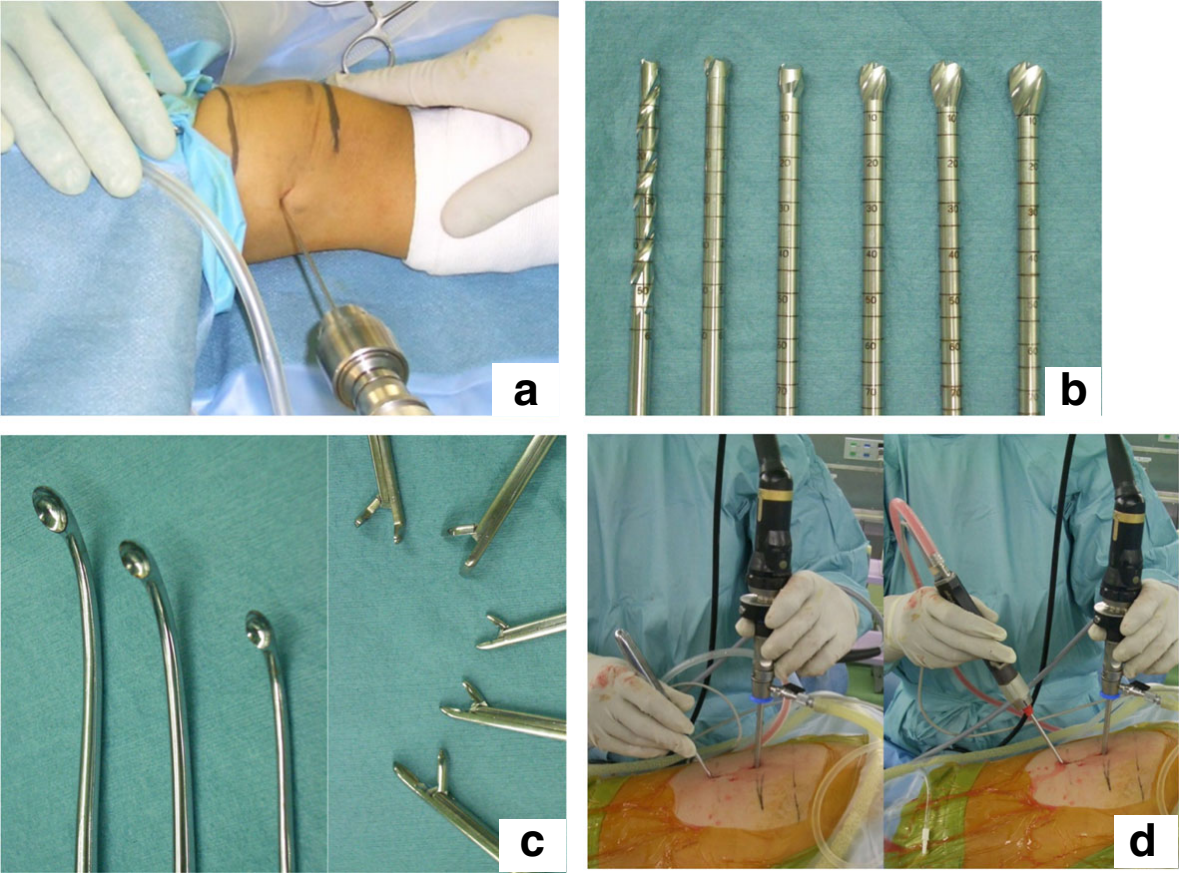
Surgical procedure and devices. **a** Penetration of the bone with a Kirschner wire. **b**Step-up cannulation up to 7–8 mm. **c** The device used for curettage, including angled curettes and rongeurs. **d** Curettage performed using angled curettes and an electric shaver

**Fig. 3:**
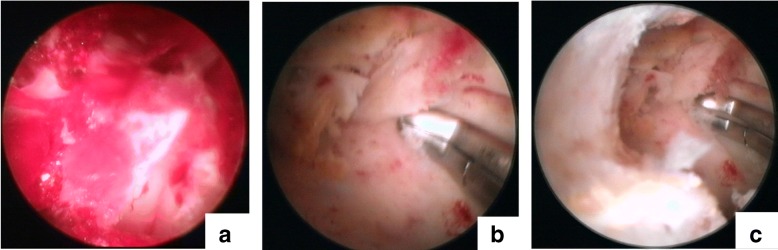
Endoscopic images. **a** The cavity of an ABC covered with blood clots and a synovial membrane. **b** The removal of neoplastic tissue using curettes. **c** Curettage was extended until normal bone was visible in the medullary cavity

Tumour location was determined using an image intensifier and marked on the skin. The position and number (2–4) of portals for the arthroscope were based on the location and size of the tumour and created via a 1-cm-long skin incision with blunt dissection. Then, the cortical bone was penetrated using Kirschner wire. The small aperture of the cortical bone was subsequently enlarged up to 7–8 mm using step-up cannulated drills (Fig. 2a). Then, an arthroscope (straight or angled) was inserted and the tumour was curetted using angled curettes, rongeurs, or an electric shaver (Fig. 2b-d). The insertion holes for the arthroscope and surgical instruments were interchanged to achieve complete curettage and provide an adequate visual field through the arthroscope. The cystic lesion was thoroughly removed until normal bone was observed in the medullary cavity (Fig. 3a-c).

Postoperatively, patients who were treated for an ABC of the lower limb were maintained until partial weight-bearing up to 50% body weight for 1 month with crutches. This was increased to two-thirds of body weight until radiological confirmation of bone healing, which typically occurred at 3 months postoperatively. Patients with an ABC on the upper limb or non-weight-bearing bone had no restrictions in activities except for contact sports until bone healing.

### Postoperative evaluation

The rate of postoperative complications and functional recovery, measured in terms of the Musculoskeletal Tumor Society Score (MSTS), was evaluated after treatment [11]. Regarding bone healing time after ESC, solid union was determined based on the criteria of Hou et al. [12], which was defined as sufficient regression of the cyst to obtain a cortical wall thick enough to prevent further fracture and allow unrestricted physical activity. Cyst consolidation at the final evaluation was classified based on the modified Neer classification [12, 13] (Table 1).

**Table 1:** Modified Neer classification

Classification	Description	Details
A	Healed	Cyst filled with new bone with small radiolucent area (< 1 cm)
B	Healed with a defect	Radiolucent area (< 50% diameter) with enough cortical thickness
C	Persistent cyst	Radiolucent area (≧50% diameter) with thin cortical rim
D	Recurrent cyst	Cyst reappearing in the obliterated area or in the increased residual radiolucent area

### Statistical analysis

Risk factors for recurrence were evaluated using the Kaplan-Meier analysis and the log-rank test. Anonymised radiographical images were evaluated by an orthopaedic surgeon (H.A.) and a radiologist (T.G.) for double-blind confirmation of the classification. Then, the kappa value was calculated to evaluate inter-observer errors regarding the morphological appearance of the bone. For statistical analysis of the categorical data, a chi-squared analysis was used. A *p*-value < 0.05 was considered significant for all analyses. Measured variables were presented as mean and standard deviation or median with range, as appropriate, for the data distribution. All statistical analyses were conducted using SPSS version 24 (IBM, Chicago, IL).

## Results

**Fig. 4:**
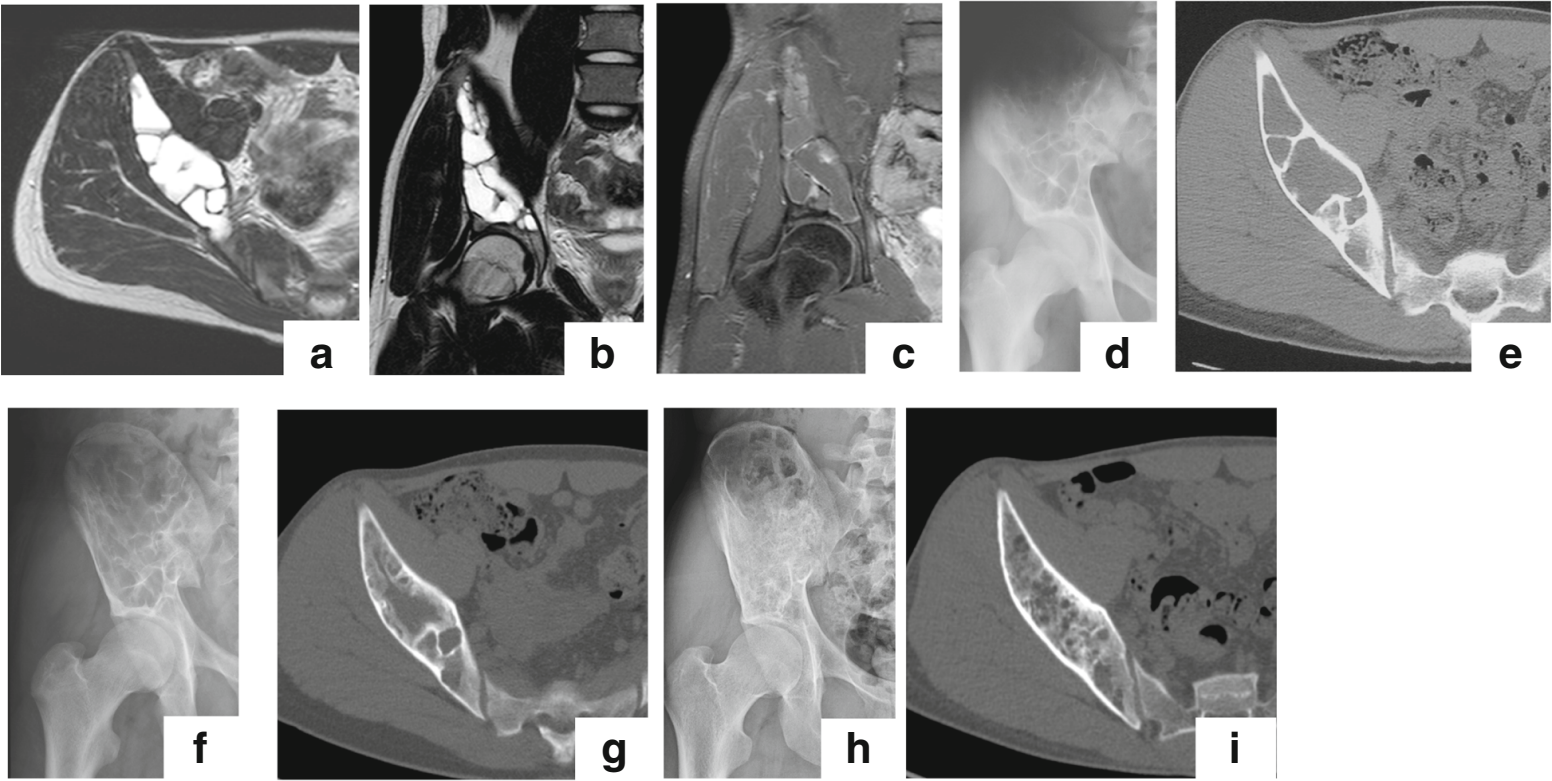
Typical case of a suspected ABC treated with ESC. Magnetic resonance (MR) images of a 16-year-old boy who presented with bruising of his hip sustained during contact sports participation: (**a**) axial T2; (**b**) coronal T2-weighted; and (**c**) coronal T1-weighted enhanced with gadolinium (Gd). The plain radiograph is shown in (**d**). The preoperative computed tomography (CT) image (**e**) shows the bone lesion with cortical thinning extending from the iliac wing to the periacetabular area. Plain radiographs after surgery (**f**). CT image at 6 months after ESC (**g**) shows remodeling of the cystic lesion by cancellous bone. At 6 years after ESC (**h**), the pelvic bone was fully remodeled despite a residual cyst and without any symptoms (class B) (**i**)

Among the 30 patients included in our study group, ESC was performed using an average of 2.3 portals (4 portals in 3 cases; 3 portals in 4 cases; and 2 portals in 23 cases). The number of portals depended on the size of the lesion. The median operative time was 107.8 (range, 47.0–197.0 min), with a median intraoperative bleeding volume of 34.2 mL (range, near 0–354 mL), which was estimated from the total amount of irrigation fluid. Images for a typical case are shown in Fig. 4.

### Recurrence rate after ESC

During the follow-up period, tumour recurrence was identified in 3 cases (3/30; 10%), with a curative outcome achieved in all 3 cases with repeated ESC or open curettage. The log-rank analysis (Table 2) showed that age < 10 years (*p* = 0.004) and tumour involvement of the physis (*p* = 0.01) were associated with tumour recurrence.

**Table 2:** Variables associated with ABC tumour recurrence

Variable	Number of cases (*N* = 30)	Recurrence	*P*-value
Age, y			0.004
< 10	8	3	
≧10	22	0	
Sex			0.776
Male	18	2	
Female	12	1	
Location			0.949^a^
Tubular bone	19	2	
Flat bone	7	1	
Short bone	3	0	
Sesamoid bone	1	0	
Pathologic fracture			0.220
Yes	4	1	
No	26	3	
Contact with physis			0.010
Yes	10	3	
No	20	0	
Maximum length of tumour			0.966
< 50 mm	11	1	
≧50 mm	19	2	

^a^Pooled over strata comparison

### Healing after ESC

The average time to solid union after ESC was 3.2 (SD, 1.7) months. The modified Neer classifications of the final status after ESC for all 30 cases were as follows: 21 cases with class A-B (healed cyst) and 9 cases with class C (persistent cyst) (assessed by H.A.). The kappa value of intra-observer reliability was substantial (0.76; *p* = 0.001; the differences of the 2 observers are provided in Additional file 1). Although tumour size was not significantly associated with the residual tumour, there was a predilection for cyst residue (class C) with large tumours (> 50 mm; *p* = 0.09; chi-squared analysis). For all class C cases, the cystic lesion did not increase during the follow-up period. These patients were carefully monitored at each follow-up visit. The diameter of the cyst remained stable in all cases over several years until the last follow-up.

### Complications and function after ECS

Almost all patients recovered excellent function after ESC (MSTS = 30), with the exception of 1 patient who reported persisting pain due to a lumbar disc herniation. Tumour recurrence was identified in 3 cases, with 1 progressing to a postoperative fracture after the third recurrence. Transient radial nerve palsy was identified in 1 case. There were no cases of infection, intraoperative fracture, or deformity.

## Discussion

In this study, we reported the surgical and clinical outcomes of a relatively large case series of patients with an ABC treated with ESC. Open curettage and reconstruction of the defect with a bone graft, as described by Jaffe et al. [14], remain the mainstay of modern treatment of ABCs [4], with the rate of tumour recurrence ranging between 40 and 90% [6]. Several procedures have been developed as options for the eradication of micro-residue tumours to improve local control and lower the rate of recurrence, including the use of high-speed burring or chemical treatment (such as the use of liquid nitrogen or polymethylmethacrylate) with or without thermal effects [15–17]. However, the exudate of these agents from the surgical site may damage the surrounding soft tissues. Therefore, endoscopy-guided surgery was proposed as an alternative treatment. Endoscopy provides the advantage of accurately assessing tumour resection via direct examination of the bone marrow cavity for complete removal of the cyst (when possible). Direct visualization also avoids blind spots or excessive curettage, which may result in an intraoperative fracture or brittleness of the treated bone [18, 19]. Furthermore, because endoscopy can be performed via small port holes requiring only small incisions, the procedure is aesthetically superior and less invasive compared to open procedures. Despite the prolonged operation time due to the preparation of holes and careful piece-by-piece curettage to avoid excessive curettage or remaining tumour, ESC might be a good alternative for the treatment of benign bone tumours.

ESC can be applied to other types of tumours. Since the early 1990s, when minimally invasive approaches were preferred, we have performed ESC for the treatment of enchondromas, unicameral bone cysts, and chondroblastomas [20–22]. Stricker introduced the extra-articular approach for the treatment of chondroblastoma arising in the epiphysis without violating the joint surface and recurrence [23]. Moreover, Errani, et al. reported their experience with the treatment of chondroblastoma arising in the vicinity of the knee joint and introduced ESC as an approach that allowed as much visualisation as possible of the residual tumour and avoided violating the joint surface [24].

ESC aims to achieve complete tumour resection and to facilitate new bone formation by direct stimulation of cysts. Recently, Reddy et al. [8] described curopsy, which is defined as percutaneous limited curettage at the time of biopsy, as a viable approach to resecting the membrane lining various quadrants of the cyst. They reported a tumour recurrence rate of 18.6% and time to bone healing of 9.6 weeks. Our results were comparable to these (10% recurrence rate after ESC). Similar to biopsy or curopsy, we postulated that ESC had a role in stimulating bone healing after penetration of small holes and was effective for removing tumour cells lining the cyst walls. In general, the healing process after biopsy of an ABC can be slow or rapid [25]. Slow healing after biopsy is a good indication for subsequent surgery to prevent further tumour progression or pathologic fracture. In contrast, rapid healing after biopsy is an indication to delay or postpone surgery to prevent unnecessary complications. Therefore, it is important to note that ABCs present with a broad spectrum of biological aggressiveness; therefore, ESC might fail in the case of aggressive lesions [26]. However, in our case series, we did not observe a rapid increase in osteolytic lesions after ESC; therefore, insistent curettage in the cavity by ESC is possibly important to achieve total removal of the resident tumour.

We excluded cases in which ESC might have lessened the strength of the bone during the postoperative period prior to bone healing. This decision was based on our prior experience during our early period of performing ESC, when we experienced 2 cases of postoperative fracture in patients in whom ESC was used for to treat a unicameral bone cyst. Currently, it is not possible to predict pathological fractures with benign bone tumours in paediatric patients [27]. Kaelin et al. [28] proposed the use of a cyst index (area of the cyst on a plain radiograph divided by the square of the diameter of the bone diaphysis at its tubular portion) to predict the risk of a pathological fracture, with a cutoff index of > 4 cysts identified for the humerus and a cutoff index of > 3.4 cysts for the femur being predictive of postoperative fractures. However, the positive predictive value of this index was limited and had a high false-positive return. Similar to the cyst index, Mirel’s score, which was originally introduced for the management of metastatic tumours and calculated as the sum of each value for the tumour position, pain, and the lesion/cortex ratio (the ratio of the maximum width of the lesion to the bone), is a practical method of predicting postoperative fractures that provides an indication for preemptive internal fixation [29]. We considered a lesion/cortex ratio of > 2/3 in the trochanteric region as a contraindication to ESC. Therefore, we chose to perform preemptive fixation in 2 cases with a cystic lesion of the trochanter due to concerns regarding severe functional restriction associated with pathological fractures of the trochanteric region of the femur and the patient anxiety associated with such an impairment. The validity of this strategy must be verified in further research.

Open growth plates [30] and the proximity of the bone cyst to the epiphysis [31] have been identified as risk factors for local recurrence. In our study, we identified age < 10 years and tumour involvement of the physis as risk factors for ABC recurrence after ESC. Based on our results, we suggest that indications for ESC should be considered carefully, and that a watch-and-see strategy is recommended for patients at high risk for local recurrence and low risk for pathological fractures.

There were some limitations to our study that need to be acknowledged. First, comparisons with other standard treatments are necessary to evaluate the outcomes of ESC treatment more precisely. Second, the sample size was limited; therefore, the power of our study was not strong. Third, all diagnoses were not confirmed by a genetic approach but histological appearance. In 2004, USP6 rearrangement and/or CDH11 rearrangement were reported in the majority of primary ABC cases (69%) [32]. Because secondary ABC did not involve these rearrangements, this translocation was considered to be a useful method of determining the differential diagnosis. Therefore, the accuracy of the diagnosis might have affected the results of this study. Finally, although all procedures were performed by a single surgeon, the effects of the learning curve for the ESC procedure need to be considered. In fact, 1 case of radial nerve palsy, probably resulting from blunt trauma in the area of the radial groove during the ESC procedure, was identified during the early technical development period. Therefore, preoperative planning is essential to avoid complications with ESC, including identification of the appropriate position of the holes to avoid the neurovascular structure and determination of the appropriate timing for intervention to lower the risk for pathological fractures.

## Conclusion

To our knowledge, this is the first report of the outcomes of ESC for ABC treatment involving a relatively large case series. Nevertheless, a reliable comparison with other methods is difficult. We reported a lower recurrence rate than previous reports, and our patients recovered without long-lasting complications and achieved good functional recovery. However, further investigations are necessary to validate our methods.

## Additional file

Additional file 1: Additional table. (DOCX 95 kb)

## Abbreviations

ABC: Aneurysmal bone cyst
ESC: Endoscopic curettage
SD: Standard deviation
