# Supplementary material for: Treatment of aneurysmal bone cysts using endoscopic curettage
Source: BMC Musculoskelet Disord. 2018 Jul 27;19:268. doi: 10.1186/s12891-018-2176-6 (PMC6064064; doi:10.1186/s12891-018-2176-6)
Supplement: Supplementary file 1 — Additional table. (DOCX 95 kb) [file 12891_2018_2176_MOESM1_ESM.docx]

| **No.** | **Position** | **Sex** | **Age (years)** | **Maximum diameter**  **(mm)** | **Physis** | **Contact with physis** | **Follow-up (month)** | **Recurrence**  **/Timing (month)** | **Treatment for recurrent lesion** | **Final status**  **(observed by H.A.)** | **Final status**  **(observed by T.G.)** | **ISOLS/MSTS score (out of 30)** | **Complication** |
| --- | --- | --- | --- | --- | --- | --- | --- | --- | --- | --- | --- | --- | --- |
| 1 | Humerus | M | 9.4 | 51 | Open | Yes | 104.4 | Yes  /8.0,24.1, 55.1 | 1, ESC; 2, ESC; 3, OC and ABG | A | B | 30 | Pathological fracture after 3^rd^ recurrence |
| 2 | Humerus | F | 12.5 | 68 | Open | No | 45.2 | No |  | C | C | 30 | Transient radial nerve palsy |
| 3 | Humerus | M | 8.9 | 50 | Open | Yes | 19.0 | No |  | C | C | 30 |  |
| 4 | Humerus | F | 24.5 | 120 | Closed | No | 31.2 | No |  | C | C | 30 |  |
| 5 | Humerus | F | 9.1 | 126 | Open | No | 60.8 | No |  | A | A | 30 |  |
| 6 | Humerus | M | 15.8 | 66 | Open | Yes | 57.5 | No |  | B | C | 30 |  |
| 7 | Humerus | M | 11.4 | 120 | Closed | Yes | 24.3 | No |  | C | C | 30 |  |
| 8 | Humerus | M | 20.3 | 58 | Closed | No | 15.9 | No |  | B | B | 30 |  |
| 9 | Humerus | F | 30.9 | 88 | Closed | No | 33.3 | No |  | C | C | 30 |  |
| 10 | Humerus | M | 32.0 | 75 | Closed | No | 149.4 | No |  | A | A | 30 |  |
| 11 | Humerus | F | 18.0 | 160 | Closed | No | 96.8 | No |  | C | B | 30 |  |
| 12 | Humerus | M | 17.4 | 73 | Closed | No | 36.0 | No |  | A | A | 30 |  |
| 13 | Tibia | F | 8.5 | 63 | Open | Yes | 34.4 | No |  | B | B | 30 |  |
| 14 | Tibia | M | 10.4 | 39 | Open | Yes | 37.2 | No |  | A | A | 30 |  |
| 15 | Tibia | M | 13.1 | 49 | Open | No | 49.7 | No |  | A | A | 30 |  |
| 16 | Tibia | M | 5.8 | 47 | Open | Yes | 58.1 | No |  | A | A | 30 |  |
| 17 | Femur | M | 8.0 | 70 | Open | Yes | 88.0 | Yes/ 42.3 | ESC | A | A | 30 | Recurrence |
| 18 | Femur | F | 16.9 | 40 | Closed | No | 61.3 | No |  | C | C | 30 |  |
| 19 | Metatarsal bone of second toe | M | 31.3 | 30 | Closed | No | 71.9 | No |  | A | A | 30 |  |
| 20 | Pelvis | F | 17 | 36 | Closed | No | 49.0 | No |  | A | B | 30 |  |
| 21 | Pelvis | F | 9.5 | 47 | Open | Yes | 112.1 | Yes/ 9.3 | OC and ABG | A | A | 28 | Recurrence |
| 22 | Pelvis | M | 9.6 | 63 | Open | No | 106.6 | No |  | C | C | 30 |  |
| 23 | Pelvis | F | 14.7 | 52 | Closed | No | 59.8 | No |  | B | B | 30 |  |
| 24 | Pelvis | F | 14.3 | 108 | Closed | No | 30.7 | No |  | B | B | 30 |  |
| 25 | Pelvis | M | 16.5 | 108 | Closed | No | 70.8 | No |  | B | B | 30 |  |
| 26 | Pelvis | M | 17.4 | 40 | Closed | No | 10.8 | No |  | B | A | 30 |  |
| 27 | Calcaneous | M | 40.0 | 34 | Closed | No | 10.8 | No |  | B | B | 30 |  |
| 28 | Calcaneous | M | 31.7 | 67 | Closed | No | 144.2 | No |  | C | C | 30 |  |
| 29 | Naviculae | F | 16.6 | 23 | Closed | No | 17.3 | No |  | B | B | 30 |  |
| 30 | Patella | M | 29.2 | 32 | Closed | No | 33.4 | No |  | B | C | 30 |  |

The final status was classified using the modified Neer classification. For all patients in group C, the cystic lesion was stable in size until the last follow-up. F, female; M, male; ESC; endoscopic curettage; OC, open curettage; ABG, artificial bone grafting. Patients 12 and 28 underwent open curettage at other institutions and were examined on a consultation basis at our hospital.
